# Spectris ^TM^ non‐invasive neuromodulation in Alzheimer’s disease

**DOI:** 10.1002/alz70859_100579

**Published:** 2025-12-25

**Authors:** Ralph Kern, Lily Lee, Robbert Zusterzeel, Alexandra Konisky, Evan Hempel, Julia M Leach, Chandran V Seshagiri, Mihaly Hajos, Christian Howell

**Affiliations:** ^1^ Cognito Therapeutics, Cambridge, MA USA; ^2^ Yale University School of Medicine, Malden, MA, MA USA

## Abstract

**Background:**

Cognito Therapeutics is advancing a convenient, at‐home neuromodulation treatment option that leverages non‐invasive audiovisual stimulation to modify the biology and clinical course of Alzheimer’s disease. The OVERTURE (NCT03556280) randomized (RCT) and open label extension (OLE) clinical trials evaluated our non‐invasive device (Spectris^TM^) in participants with mild‐moderate Alzheimer’s disease (AD). The almost fully enrolled HOPE pivotal trial (NCT05637801) is being conducted across 60 US clinical trial sites in mild‐moderate Alzheimer’s disease. We will discuss the impact Spectris^TM^ may have on patients, their families, and the broader health system to address the important unmet need in Alzheimer’s disease.

**Method:**

The OVERTURE RCT compared one‐hour, daily, at‐home, active vs. sham treatment for six months (2:1 randomization). The OVERTURE OLE continued open‐label treatment in RCT completers for an additional 12 months. The ongoing HOPE study is evaluating Spectris^TM^ in a 12‐month RCT and 12‐month OLE (NCT06245031) in participants with mild‐moderate AD.

**Result:**

OVERTURE RCT and OLE clinical and MRI biomarker results will be reviewed and discussed. We will compare the OVERTURE and HOPE study designs and baseline characteristics as well as key insights gained from the evaluation of a daily, at‐home, non‐invasive neuromodulation therapy in mild‐moderate Alzheimer’s participants.

**Conclusion:**

Spectris^TM^ potentially offers a safe, effective and cost‐effective treatment option and may expand the range of treatment options available for patients with Alzheimer’s disease.

